# Safer Prescribing and Care for the Elderly (SPACE): a pilot study in general practice

**DOI:** 10.3399/bjgpopen18X101594

**Published:** 2018-07-11

**Authors:** Katharine A Wallis, C Raina Elley, Simon Moyes, Ngaire Kerse

**Affiliations:** 1 GP and Senior Lecturer, Department of General Practice and Primary Health Care, Faculty of Medical and Health Sciences, University of Auckland, Auckland, New Zealand; 2 GP and Associate Professor, Department of General Practice and Primary Health Care, Faculty of Medical and Health Sciences, University of Auckland, Auckland, New Zealand; 3 Data Manager/Analyst, Department of General Practice and Primary Health Care, Faculty of Medical and Health Sciences, University of Auckland, Auckland, New Zealand; 4 GP and Head of School, Department of General Practice and Primary Health Care, Faculty of Medical and Health Sciences, University of Auckland, Auckland, New Zealand

**Keywords:** General practice, multimorbidity, prescriptions, polypharmacy, drug related side-effects and adverse reactions

## Abstract

**Background:**

High-risk prescribing places patients at increased risk of adverse drug events (ADEs). High-risk prescribing and ADE hospitalisations are increasingly common as people are living longer and taking more medicines for multiple chronic conditions. The Safer Prescribing and Care for the Elderly (SPACE) intervention is designed to foster patient engagement in medicines management and prompt medicines review.

**Aim:**

To pilot the SPACE intervention in preparation for a larger cluster randomised controlled trial (RCT).

**Design & setting:**

A pilot study in two general practices. Study participants were all patients at increased risk of an adverse drug reaction (ADE) from non-steroidal anti-inflammatory drugs (NSAIDs) and/or antiplatelet medicines. The primary outcome was the proportion of participants receiving high-risk prescribing at 6 months and 12 months compared with baseline.

**Method:**

The SPACE intervention comprised automated practice audit to identify and generate for each GP a list of patients with high-risk prescribing for these medicines; an outreach visit by clinical advisory pharmacist to deliver education and to go through with each GP their list of at-risk patients and indicate in a tick-box the intended action for each patient; and a mail-out from GPs to selected patients containing a medicines information brochure and a letter encouraging patients to discuss their medicines when they next see their GP.

**Results:**

SPACE can be delivered within existing primary care infrastructure. The rate of high-risk prescribing was reduced at 6 months following the delivery of the intervention, but these improvements were not evident at 12 months.

**Conclusion:**

SPACE prompts medicines review and shows promising signs of supporting safer prescribing in general practice in the short term. A randomised trial of SPACE started in 2018.

## How this fits in

High-risk prescribing places patients at increased risk of ADEs and is increasingly common. The SPACE intervention combines practice prescribing audit and feedback to doctors with a mail-out to at risk patients to prompt medicines review. SPACE can be delivered within existing primary care infrastructure. Findings from this pilot study suggest SPACE supports safer prescribing in routine general practice in the short term; however a randomised trial is needed to confirm this.

## Introduction

High-risk prescribing places patients at increased risk of an ADE. ADEs result in many avoidable hospital admissions every year, disrupting people’s lives and draining precious health resources.^1–9^ The single greatest predictor for high-risk prescribing and ADEs is the number of medicines a person is taking.^10^ High-risk prescribing and ADEs are increasingly common as more people are living longer and taking more medicines for multiple chronic conditions. Commonly prescribed medicines account for most ADE admissions, notably NSAIDs, antiplatelet medicines, and anticoagulants, which together account for one-third of all ADE admissions.^3,4^ Most ongoing prescribing occurs in general practice.

Safe prescribing entails regular review of medicines, and stopping or starting medicines as indicated. High-risk prescribing may be justified by the individual circumstances of a patient, but regular review of medicines is important to ensure ongoing safety. There is good evidence to guide safe prescribing, but a gap remains between existing evidence and current prescribing practice. Translating evidence into practice is difficult. There are many barriers to regular medicines review in routine general practice.^11^ The large variation in prescribing between practices and regions suggests room for improvement.^12–14^

The scale of the problem of high-risk prescribing and avoidable ADEs has prompted searches internationally for ways to support safer prescribing in general practice.^15^ There is good evidence that audit and feedback can change practice, especially when used in ongoing quality improvement programmes that also include education and incentives.^15–24^ For example, the pharmacist-led information technology intervention for medication error (PINCER) trial in England used a pharmacist to deliver educational outreach and feedback to GPs;^20^ the Data-driven Quality Improvement in Primary Care (DQIP) trial in Scotland used educational outreach, informatics to facilitate medicines review and provide regular feedback, and financial incentives;^18,23^ and the Optimizing Prescribing for Older People in Primary Care, a cluster-randomised controlled trial (OPTI-SCRIPT trial) in the Republic of Ireland used academic detailing by a pharmacist and a web-guided medication review.^24,25^ There is also the Australian Veterans’ Medicines Advice and Therapeutics Education Service (MATES) quality improvement programme that combines practice prescribing audits and patient-based feedback and education to doctors, with a practice mail-out to at-risk patients.^19^

The most effective, cost-effective, and practical approach to safer prescribing in New Zealand is not yet known. In New Zealand, general practices are organised into primary health organisations (PHOs) that deliver most quality improvement initiatives. Some PHOs employ clinical advisory pharmacists to support prescribing. GPs report suffering from alert fatigue, and that educational support and financial incentives would support safer prescribing.^11^ However, financial incentives may not be sustainable and can have unintended consequences. Based on the existing knowledge base and qualitative findings, the MATES intervention was adapted to the New Zealand primary care context creating the SPACE intervention.^26^

SPACE comprises an automated practice prescribing audit to identify and generate for each doctor a list of patients with high-risk prescribing; an outreach visit from a clinical advisory pharmacist to deliver a group educational session, and to meet one-on-one with each doctor to go through their list of patients and to indicate in a tick-box an intended action for each patient (no change/change prescribing/letter to patient); and an automated practice mail-out to selected patients of a medicines information brochure and a letter encouraging patients to discuss their medicines when they next see their doctor. SPACE was designed to prompt medicines review and support safer prescribing in routine practice. All prescribing decisions are made as usual by the doctor together with the patient. SPACE differs from MATES in that it is not part of an ongoing quality improvement programme, targets all doctors and at-risk patients in a practice, and has no national call centre. SPACE does not use financial incentives or provide regular updates and comparators. The novel aspect of SPACE is the attempt to engage patients through a practice mail-out. The utility and acceptability of SPACE have been confirmed.^26^

The authors worked with one PHO to pilot the SPACE intervention in two practices in preparation for a larger cluster RCT. The specific objectives of the pilot study were to optimise delivery of the SPACE intervention; test data management processes and outcome measures; and to assess whether SPACE reduced the rate of high-risk prescribing.

## Method

The high-risk prescribing topic chosen for this pilot study was NSAIDs and antiplatelet medicines.^21^ High-risk prescribing of these medicines has been studied in previous trials.^18^

### Setting & participants

The setting was two general practices from one PHO in Auckland, New Zealand. Eligibility was restricted to practices using MedTech practice management software. The pharmacist recruited two practices, one medium and one small, from the PHO network of member practices. Since patients may receive prescriptions from any GP in a practice, informed consent was obtained from all GPs in each participating practice.

Study participants were all patients who were vulnerable at baseline; that is, those patients at increased risk of an ADE when prescribed NSAIDs or antiplatelet medicines as set out in Box 1. The study was focused on older adults, but not restricted to this group as younger patients who fitted the categories not dependent on age, such as history of peptic ulcer ever or renal impairment (most recent estimated glomerular filtration rate [eGFR] <60), were included. A computer query was run over each practice database of enrolled patients to identify patients fulfilling eligibility criteria for ‘vulnerability’ to ADE according to the demographic, clinical, and prescribing criteria described in Box 1. A second database query identified those ‘vulnerable’ individuals who also received ‘high risk prescribing’ in the previous 14 weeks according to prescribing data (Box 1). These queries can be run remotely by the PHO or by Dr Info, an organisation contracted by the practices to run clinical audits for the practices’ benefit. Individual patient consent to participation was not sought because all treatment decisions were made as usual by the GP together with the patient, and because outcomes data were collected in routine patient care and were anonymised prior to extraction for analysis and linking.

**Box 1.** Categories of vulnerable patients and high-risk prescribing of NSAIDs and antiplatelet medicines^17^

Type of adverse drug event	Vulnerable patients (at increased risk of ADE)	High-risk prescribing^a^
Gastrointestinal bleed	Prior peptic ulcer ever	NSAID or aspirin without gastroprotection, in patient with prior peptic ulcer
	Aged ≥75 years	NSAID without gastroprotection, in patient ≥75 years
	Aged ≥65 years prescribed aspirin	NSAID without gastroprotection, in patient ≥65 years taking aspirin
		Clopidogrel without gastroprotection, in patient ≥65 years taking aspirin
	Prescribed oral anticoagulant	NSAID without gastroprotection, in patient taking an oral anticoagulant
		Aspirin or clopidogrel without gastroprotection, in patient taking an oral anticoagulant
Renal impairment	Prescribed both renin-angiotensin system blocker and diuretic	NSAID, in patient taking both renin-angiotensin system blocker and diuretic
	Chronic kidney disease (most recent eGFR <60)	NSAID, in patient with chronic kidney disease (eGFR <60)
Cardiac failure	Heart failure ever	NSAID, in patient with history of heart failure

^a^Medicines prescribed within the previous 14 weeks. ADE = adverse drug event. eGFR = estimated glomerular filtration rate. NSAID = nonsteroidal anti-inflammatory drug.

### Intervention

The SPACE intervention comprises:

1. a practice audit to identify and generate for each GP a list of patients with high-risk prescribing of NSAIDs and/or antiplatelet medicines;
2. an outreach visit by a clinical advisory pharmacist to provide:
  1. a group educational session to GPs about the prescribing topic;
  2. a one-on-one meeting with GPs to go through their list of at-risk patients and to indicate in a tick-box the intended action for each patient (no action/review medicines/mail-out to patient); and
3. a practice mail-out to selected patients containing a medicines information brochure and a letter encouraging patients to discuss their medicines when they next see their doctor.

### Outcome measures

The outcome measures were process outcomes and high-risk prescribing outcomes. Process outcomes included the success and time taken to deliver the intervention; and the data extraction, anonymisation, and linking over time. High-risk prescribing outcome measures were the proportion of study participants receiving high-risk prescribing of NSAID and/or antiplatelet medicines at 6 months and 12 months compared with baseline. The primary outcomes were the proportion of study participants (patients who were vulnerable at baseline) who received high risk prescribing of NSAIDs and/or antiplatelet medicines in the 14 weeks prior to each time-point, according to the definitions listed in Box 1.

The secondary high-risk prescribing outcome measures included:

1. the proportion of study participants at increased risk of gastrointestinal ADEs receiving high-risk prescribing of NSAID and/or antiplatelet medicines;
2. the proportion of study participants at increased risk of renal ADEs receiving high-risk prescribing of NSAID medicines;
3. the proportion of study participants at increased risk of cardiac ADEs receiving high-risk prescribing of NSAID medicines; and
4. overall practice rate of high-risk prescribing of NSAIDs and antiplatelet medicines. This included vulnerable patients who were newcomers to the practice at 6 or 12 months, and practice patients who were not previously vulnerable (at increased risk of an ADE) but who became so.

### Analyses

Data on patients were anonymised prior to the data leaving the practice using an encrypted unique patient identifier, the National Health Index (NHI) number, to allow linking over time. Demographic and clinical characteristics of the study practices and patients were described. Outcomes at 6 months and 12 months were compared with baseline, using repeated measures generalised linear mixed models. *P*-values of 0.05 were considered statistically significant. Sensitivity analyses used last value carried forward for those participants who were lost for follow-up.

## Results

### Practice and patient characteristics

Practice A included six GPs with 7944 enrolled patients, and Practice B had one GP and 2500 enrolled patients. From the total of 10 444 enrolled patients, 870 (8.3%) individual patients were at increased risk of ADE related to NSAIDs and/or antiplatelet medicines (vulnerable patients) and comprised the study population. Table 1 describes the demographic characteristics of the study population.

**Table 1.** Characteristics of study population (patients who were vulnerable at baseline, that is at increased risk of an adverse drug event with non-steroidal anti-inflammatory drugs and/or antiplatelet medicines^a^) from the two participating general practices

	General practice A (registered patients: *n *= 7944)	General practice B (registered patients: *n *= 2500)	Total
Vulnerable patients^a^, *n* (%)	668 (8.6)	202 (8.1)	870 (8.9)
Age, mean (SD)	74.9 (11.2)	73.2 (12.3)	74.5 (11.4)
Female, *n* (%)	392 (58.7)	96 (47.5)	488 (56.1)
**Ethnicity, *n* (%):**			
New Zealand European	321 (48.1)	157 (77.7)	478 (54.9)
Other European	253 (37.9)	23 (11.4)	276 (31.7)
New Zealand Maori	7 (1.0)	4 (2.0)	11 (1.3)
Pasifika	5 (0.7)	1 (0.5)	6 (0.7)
East Asian	29 (4.3)	6 (3.0)	35 (4.0)
Indian	12 (1.8)	7 (3.5)	19 (2.2)
Other	41 (6.1)	4 (2.0)	45 (5.2)
Number of long-term medicines, mean (SD)	4.07 (1.86)	1.35 (0.56)	3.44 (2.01)

^a^Patients with one or more risk factor for gastrointestinal, renal, or cardiac adverse effects when prescribed NSAID and/or antiplatelet medicines. SD = standard deviation.

The practice audits identified 70 individual patients with high-risk prescribing (8.0% of vulnerable patients). Some patients fulfilled more than one of the high-risk prescribing criteria set out in Box 1.

### Intervention delivery

The PHO clinical advisory pharmacist delivered one-on-one feedback and education to all seven doctors in the two practices. In the group practice, the pharmacist delivered education about the prescribing topic in a group session, which took about 30 minutes, followed by the one-on-one session with each doctor to go through their list of at-risk patients, which took about 15 minutes each.

Overall, the GPs selected 29 of the 70 patients with high-risk prescribing (41.4%) to receive the mail-out. The main reason GPs decided not to send the mail-out to patients was because the high-risk prescribing had already ceased (for example, the NSAID prescription had been for only a short course); other reasons included the patient had transferred to another practice, the NSAID prescription had been for gel, the renal function had improved and eGFR was now >60, and/or because the GP was not convinced the prescribing was high risk.

### Data extraction, encryption, and linkage processes

The automated data extraction and encryption processes worked well. There was some refinement in data extraction methods between baseline and 6 months, particularly around the eGFR (changed from ‘ever’ to ‘latest’ eGFR <60). This ensured that prescribing was deemed ‘high risk' only due to the latest eGFR being <60, and not based on a historically low eGFR. There were 32/870 (3.7%) patients lost to follow-up between baseline and 6 months who were included in the study population due to a low eGFR at baseline, of which only three of 32 were found to have high-risk prescribing where it could not be retrospectively confirmed that the low eGFR was the most recently recorded when the prescribing occurred. This is unlikely to have made a difference to the outcomes presented below.

### High-risk prescribing outcomes

**Figure 1.**
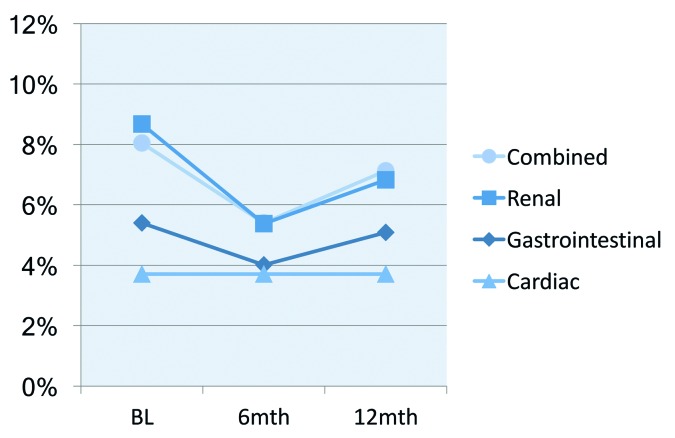
High-risk prescribing rates in study population (vulnerable at baseline) for each high-risk category and combined. BL = baseline.

At 6 months there was a significant reduction in the proportion of study participants receiving high-risk prescribing, reducing from 8.0% to 5.8% (*P*<0.05), even when all those lost to follow-up were assumed to have the same high-risk prescribing status as baseline (Table 2 and Figure 1). Changes in secondary outcomes showed similar significant improvements, except for those with congestive heart failure, as these rates were already very low at baseline (Table 2). However, at 12 months the proportion of study participants receiving high-risk prescribing had returned to baseline levels or worse. The overall practice rates of high-risk prescribing did not show a reduction at 6 months, with trends increasing at 12 months (Table 2)

**Table 2.** Rates of high-risk prescribing at baseline, and 6 and 12 months post-intervention for study population and for practice population overall

		Study population^a^				Practice population^b^	
Outcome measure	Baseline	6-month *n/N* (%)	*P*-value	12-month *n/N* (%)	*P*-value^c^	6-month *n/N* (%)	12-month *n/N* (%)
Primary outcome: high-risk prescribing	70/870 (8.0)	47/807 (5.8); 52/870 (6.0) LVCF	0.030.04	62/757 (8.2); 74/870 (8.5) LVCF	0.90.7	71/890 (8.0)	101/1026 (9.8)
**Secondary outcomes**							
High-risk prescribing among patients with any gastrointestinal risk factor	35/649 (5.4)	26/596 (4.4); 29/649 (4.5) LVCF	0.40.4	33/554 (6.0); 42/649 (6.5) LVCF	0.60.4	39/698 (5.6)	52/731 (7.1)
High-risk prescribing amongst patients with any renal risk factor	41/476^d^ (8.6)	22/444 (5.0); 25/476 (5.3) LVCF	0.0070.008	33/414 (8.0); 38/476 (8.0) LVCF	0.70.7	38/365 (10.4)	61/536 (11.4)
High-risk prescribing among patients with congestive heart failure	1/27 (3.7)	1/23 (4.4); 1/27 (3.7) LVCF	0.91.0	1/21 (4.8); 1/27 (3.7) LVCF	0.91.0	1/26 (3.9)	1/25 (4.0)

^a^Patients vulnerable at baseline; that is at increased risk of adverse drug events (ADE) when prescribed NSAID and/or antiplatelet medicines (see Table 1). ^b^All practice vulnerable patients; that is includes patients who were not vulnerable at baseline but who have become so at 6 months and/or 12 months. ^c^Compared with baseline.^d^32/870 (3.7%) patients were included as at-risk of ADE due to a low eGFR, where it could not be confirmed that the latest eGFR was still <60. LVCF = last value carried forward, which assumes that those lost to follow-up (left the practice or died) had the same high-risk prescribing status as baseline.

## Discussion

### Summary

The SPACE intervention was designed to foster patient engagement in medicines management and prompt medicines review. Results from this pilot study were encouraging in the short-term. SPACE was feasible within existing primary care structures; the data collection, encryption processes, and outcome measures worked well; and there were statistically significant improvements. There was a reduction in the rate of high-risk prescribing in the study population at 6 months post-intervention, but these improvements were not evident at 12 months.

### Strengths and limitations

This was a pilot study and there was no control group. Therefore, an RCT is needed to test whether any improvements in prescribing were due to the intervention. A cluster RCT began in 2018.^27^ Data collection methods became more refined over the 12 months, particularly around the date of eGFR, which may have influenced the inclusion criteria of 32/870 (3.7%) patients at baseline. This is unlikely to have altered results, and has led to an improved data collection and assessment method for the main trial.

### Comparison with existing literature

Findings from this pilot study suggest SPACE may have a similar effect on prescribing to that in the PINCER trial, in that the effect was greatest at 6 months’ follow-up with a slackening off by 12 months.^20^

The novel aspect of the SPACE intervention is that SPACE explicitly aims to engage patients, but GPs opted to send the mail-out to only a minority of patients. Findings from qualitative research suggest both GPs and patients find the mail-out useful and acceptable, and that the most common reason for not sending the mail-out to a patient on the list was that the high-risk prescribing had already ceased.^26^ It was not possible to refine the computer search to exclude prescriptions for short-courses or gel NSAIDs. Data were not collected on the proportion of patients who responded to the mail-out.

### Implications for research and practice

Findings from this research have informed the development of a new tool, SPACE, which can be tested as an ongoing quality improvement tool to support safer prescribing in New Zealand general practice. SPACE has the advantage of being feasible within existing primary care structures. If effective, PHOs could roll out and use SPACE regularly in practices to support safer prescribing. Similar large-scale approaches have been used successfully elsewhere.^18–20,24^

Results from this pilot study suggest that audit, education, and patient prompting may help support safer prescribing of NSAIDs and antiplatelet medicines. However, findings also suggest this effect may be short-lived, suggesting the intervention may need to be used regularly in practices to sustain change. Despite the drop-off in effect at 12 months, it was decided to confirm the effect of this one-off brief intervention in a large cluster RCT, which commenced early 2018. In practice, SPACE could be used repeatedly, addressing the same or a different prescribing topic every few months. Ultimately, the aim is to reduce the incidence of related ADEs, particularly those leading to hospitalisation or death, which will also be tracked in the cluster RCT, and particularly among older adults who make up the majority of vulnerable patients.

## References

[1] Taché SV, Sönnichsen A, Ashcroft DM (2011). Prevalence of adverse drug events in ambulatory care: a systematic review. Ann Pharmacother.

[2] Budnitz DS, Lovegrove MC, Shehab N (2011). Emergency hospitalizations for adverse drug events in older Americans. N Engl J Med.

[3] Pirmohamed M, James S, Meakin S (2004). Adverse drug reactions as cause of admission to hospital: prospective analysis of 18 820 patients. BMJ.

[4] Howard RL, Avery AJ, Slavenburg S (2007). Which drugs cause preventable admissions to hospital? A systematic review. Br J Clin Pharmacol.

[5] Meier F, Maas R, Sonst A (2015). Adverse drug events in patients admitted to an emergency department: an analysis of direct costs. Pharmacoepidemiol Drug Saf.

[6] Guthrie B, McCowan C, Davey P (2011). High risk prescribing in primary care patients particularly vulnerable to adverse drug events: cross sectional population database analysis in Scottish general practice. BMJ.

[7] Wallis KA (2015). Learning from no-fault treatment injury claims to improve the safety of older patients. Ann Fam Med.

[8] Dovey SM, Leitch S, Wallis KA (2017). Epidemiology of patient harms in New Zealand: Protocol of a General Practice records review study. JMIR Res Protoc.

[9] Thomsen LA, Winterstein AG, Søndergaard B (2007). Systematic review of the incidence and characteristics of preventable adverse drug events in ambulatory care. Ann Pharmacother.

[10] Fried TR, O'Leary J, Towle V (2014). Health outcomes associated with polypharmacy in community-dwelling older adults: a systematic review. J Am Geriatr Soc.

[11] Wallis KA, Andrews A, Henderson M (2017). Swimming against the tide: primary care physicians' views on deprescribing in everyday practice. Ann Fam Med.

[12] Love T, Ehrenberg N (2014). Variation and improving services: case studies and key questions. http://www.hqsc.govt.nz/assets/Health-Quality-Evaluation/PR/Variation-case-studies-and-key-questions-May-2014.pdf.

[13] Health Quality & Safety Commission New Zealand (2015). Polypharmacy in people aged 65 and over. http://www.hqsc.govt.nz/our-programmes/health-quality-evaluation/projects/atlas-of-healthcare-variation/polypharmacy-in-older-people/.

[14] Tomlin AM, Gillies TD, Tilyard MW (2016). Variation in the pharmaceutical costs of New Zealand general practices: a national database linkage study. J Pub Health.

[15] Patterson SM, Cadogan CA, Kerse N (2014). Interventions to improve the appropriate use of polypharmacy for older people. Cochrane Database Syst Rev.

[16] Ivers N, Jamtvedt G, Flottorp S (2012). Audit and feedback: effects on professional practice and healthcare outcomes. Cochrane Database Syst Rev.

[17] Clyne B, Fitzgerald C, Quinlan A (2016). Interventions to address potentially inappropriate prescribing in community-dwelling older adults: a systematic review of randomized controlled trials. J Am Geriatr Soc.

[18] Dreischulte T, Donnan P, Grant A (2016). Safer prescribing — a trial of education, informatics, and financial incentives. N Engl J Med.

[19] Roughead EE, Kalisch Ellett LM, Ramsay EN (2013). Bridging evidence-practice gaps: improving use of medicines in elderly Australian veterans. BMC Health Serv Res.

[20] Avery AJ, Rodgers S, Cantrill JA (2012). Sheikh A. A pharmacist-led information technology intervention for medication errors (PINCER): a multicentre, cluster randomised, controlled trial and cost-effectiveness analysis. Lancet.

[21] Thabane L, Ma J, Chu R (2010). A tutorial on pilot studies: the what, why and how. BMC Med Res Methodol.

[22] Duerden M, Avery T, Payne R (2013). Polypharmacy and medicines optimisation: making it safe and sound.

[23] Guthrie B, Kavanagh K, Robertson C (2016). Data feedback and behavioural change intervention to improve primary care prescribing safety (EFIPPS): multicentre, three arm, cluster randomised controlled trial. BMJ.

[24] Clyne B, Smith SM, Hughes CM, OPTI-SCRIPT study team (2015). Effectiveness of a multifaceted intervention for potentially inappropriate prescribing in older patients in primary care: A cluster-randomized controlled Trial (OPTI-SCRIPT study). Ann Fam Med.

[25] McCarthy C, Clyne B, Corrigan D (2017). Supporting prescribing in older people with multimorbidity and significant polypharmacy in primary care (SPPiRE): a cluster randomised controlled trial protocol and pilot. Implement Sci.

[26] Wallis K, Tuckey R (2017). Safer Prescribing and Care for the Elderly (SPACE): feasibility of audit and feedback plus practice mail-out to patients with high-risk prescribing. J Prim Health Care.

[27] Wallis KA, Elley CR, Lee A (2018). Safer Prescribing and Care for the Elderly (SPACE): protocol of a cluster randomized controlled trial in primary care. JMIR Research Protocols.

